# Effects of mind–body exercise on physical ability, mental health and quality of life in stroke patients: a systematic review and meta-analysis

**DOI:** 10.3389/fpubh.2024.1432510

**Published:** 2024-12-20

**Authors:** Jin Dong, Jinjin Chi, Desheng Wang

**Affiliations:** Physical Education Institute, Shanxi University, Taiyuan, China

**Keywords:** mind–body exercise, Tai Chi, Qigong, yoga, balance ability, quality of life, meta

## Abstract

**Purpose:**

To systematically evaluate the effects of mind–body exercise on physical ability, mental health and quality of life in stroke patients.

**Methods:**

According to the PRISMA statement, we searched Web of science, Pubmed, Embase, Sinomed, CNKI, Wanfang, and VIP databases to collect randomized controlled trials on the effects of mind–body exercise on improving balance function, motor capacity, walking function, depression and quality of life in stroke patients. The search was conducted in January 2024. Review Manager5.3 was used for statistical analysis of the data.

**Results:**

A total of 33 randomized controlled trials with a total of 1985 participants were included. The results of meta-analysis showed: Mind–body exercise had a significantly effect on balance ability [MD = 5.64, 95%CI = 4.17, 7.11, *p* < 0.00001], upper limb motor ability [MD = 6.98, 95%CI = 1.96, 12.01, *p* = 0.006 < 0.01], lower limb exercise capacity [MD = 3.55, 95% CI = 0.31, 6.78, *p* = 0.03 < 0.05], exercise capacity [MD = 7.24, 95% CI = 4.36, 10.12, *p* < 0.00001], depression [MD = −3.28, 95%CI = −3.86, −2.69, *p* < 0.00001] and quality of life [MD = 10.62, 95%CI = 5.17, 16.06, *p* = 0.0001 < 0.01]. However, mind–body exercise did not affect walking ability [MD = −1.82, 95%CI = −4.20, 0.57, *p* = 0.14 > 0.05]. The results of subgroup analysis showed: Qigong (Baduanjin) exercise for more than four weeks, 6–10 times a week, 15–40 min each time can significantly improve balance function and quality of life in stroke patients.

**Conclusion:**

Mind–body exercise can be used as a supplement therapy to conventional rehabilitation therapy, which is not only low intensity, high safety, but also because it is not limited to the site, can be accepted by most people and accelerate the rehabilitation process of stroke.

## 1 Introduction

Stroke, also known as cerebrovascular disease, refers to insufficient blood supply or bleeding in specific areas of the brain. In most cases, it can lead to cell death, functional impairment, and impaired neurological function that reflects the location and size of brain regions (1). It has become a global health challenge. Although there are great geographical differences in the epidemiological statistics of stroke, its significant characteristics include high incidence rate, morbidity, mortality and disability rates, among which the incidence of low - and middle-income countries, especially Eastern Europe and Sub Saharan Africa, is the highest (2). According to the 2019 Global Burden of Disease Study (3), stroke remains the second leading cause of death and the third leading cause of death and disability worldwide. Stroke is often accompanied by hemiplegia, depression, motor disorders, cognitive impairment, and other sequelae, which seriously affect an individual’s physical function, mental health, and quality of life.

Aerobic exercise is considered a key component of stroke rehabilitation (4). There is evidence to suggest that aerobic training is a safe intervention that can not only increase the recruitment of exercise units in stroke patients, prevent muscle atrophy, and improve their exercise ability; It can also increase the maximum oxygen uptake level of stroke patients and reduce cardiovascular risk (5, 6). In addition, resistance exercise is also considered a safe and effective tool to support post stroke recovery, which can improve upper and lower limb muscle strength, motor function, and quality of life in stroke patients (7). These exercises are all based on physical training aimed at changing the patient’s physical function. However, stroke patients also have psychological health issues, such as anxiety, depression, and sleep problems (8). About one third of patients suffer from depression after stroke, and the incidence rate of PSD in the past 2 years is between 11 and 41% (9). Therefore, we should not only focus on the physical impact of stroke patients, but also emphasize their mental recovery.

Mind–body exercise is a multifaceted form of physical activity that combines exercise sequences, respiratory control, and attention regulation (10). The characteristics of this form of exercise are deliberate slow movements, symmetrical postures, stretching and relaxation of muscles and bones, controlled breathing, and mental focus (11). It is worth noting that Tai Chi, yoga, and qigong are widely recognized as the most popular ways of mind–body exercise (12). The study by Zou et al. (11) showed that mind–body exercise has a significant impact on depression, daily living activities, and mobility in stroke patients, but has no positive impact on sleep quality. Su et al.’s research shows that three mind–body exercises, Tai Chi, Qigong, and yoga, can significantly improve the quality of life of patients. Among them, Tai Chi has shown the most comprehensive improvement in balance, limb movement function, daily life activities, and depression (13). Chen et al.’s research suggests that mind–body exercise can have a beneficial impact on balance ability in the short term (14). The above evidence indicates that mind–body exercise can not only improve the physical function of stroke patients, but also have a certain impact on their mental health.

At present, there are few literature reviews on the impact of mind–body on stroke patients, and there is a lack of recommendations for exercise prescriptions. In this study, Tai Chi, Qigong (Baduanjin) and yoga were included as mind–body exercise interventions. This article will conduct a meta-analysis to consolidate existing evidence and elucidate the rehabilitation effects of mind–body exercise on the physical function, mental health, and daily activities of stroke patients, with the aim of conducting a relatively comprehensive study on its outcome indicators. In addition, by studying the effects of different intervention methods, durations, cycles, and frequencies on patient balance function, relevant exercise prescriptions are formulated to lay a theoretical foundation for enhancing balance function in stroke patients in clinical settings.

## 2 Research methods

### 2.1 Literature search strategy

Under the guidance of PRISMA guidelines, the study was conducted using various databases including Web of Science, PubMed, Embase, Cochrane Library, CNKI, Wanfang, and VIP. The search was focused on journal articles, with the search period ending on January 26, 2024. Chinese search terms included “mind–body exercise,” “Tai chi,” “Qigong,” “yoga,” “Baduanjin” and “stroke.” English search terms include: “mind–body exercise,” “Tai Chi,” “Taiji,” “Qigong,” “Baduanjin,” “Yoga,” “Stroke,” “Cerebrovascular Accident,” “CVA,” “Cerebrovascular Apoplexy,” “Brain Vascular Accident,” “Cerebrovascular Stroke,” “Apoplexy,” “Cerebral Stroke,” “Acute Stroke,” “Acute Cerebrovascular Accident” et al. The language of the included studies were limited to English and Chinese. The search strategies used in this study for Web of Science is presented in Supplementary Table 1.

### 2.2 Eligibility criteria

#### 2.2.1 Types of studies

In this review, the types of studies included are parallel controls in randomized controlled trials (RCTs). Cross design or other types will be excluded. In addition, the participants, interventions, controls, outcome indicators, etc. of the study must meet the following requirements.

#### 2.2.2 Types of participants

The age of the participants is ≥18 years old, meeting the diagnostic criteria for stroke, and has been confirmed by CT or MRI. In the stage of stroke recovery. The participants were conscious, and had no cognitive dysfunction. Participants with severe sequelae, such as mobility impairments, were excluded.

#### 2.2.3 Types of interventions

In this review, we consider “mind–body exercise” as an intervention method for stroke patients, including Tai Chi, Qigong (Baduanjin), and yoga. The experimental group can only involve one type of mind–body exercise, and mixed exercise will be excluded.

#### 2.2.4 Types of comparators

In this review, we first excluded studies without control groups. In the control group, routine care, traditional rehabilitation training (balance function training, walking ability training, et al) and stretching will be retained. For example, a control group with tai chi or resistance exercise will be excluded.

#### 2.2.5 Types of outcomes measures

The results reported from each embedded RCT included at least one of the following outcomes:

1. Balance ability: Berg Balance Scale (BBS).
2. Motor ability: Motor Function Scale (Fugl-Meyer, FMA).
3. Walking ability: Timed Up-and-go Test (TUGT).
4. Mental health: Hamilton Depression Scale (HAMD).
5. Quality of life: Barthel Index Rating Scale (BI), Modified Barthel Index Rating Scale (MBI).

### 2.3 Literature management and data extraction

Document management using Endnote X20. Two reviewers (JC and EL) independently screened the titles and abstracts of the citations retrieved from seven electronic databases, removed duplicates, and identified eligible randomized controlled trials based on inclusion criteria. After reading the full text of the above qualified literature, the final included literature can be determined after exclusion. Two reviewers work together to resolve differences. If the discrepancy persists, a third reviewer (JD) will be invited to adjudicate.

The basic data were extracted from the included literature. The contents of literature extraction include: (1) Author and publication year; (2) Gender of subjects and sample size; (3) Age of the subject; (4) Intervention measures in the experimental group (time, frequency and method); and (5) Outcome indicators.

### 2.4 Literature bias risk assessment

Based on the Cochrane risk Bias assessment tool (15), Review Manager 5.3 software was used to conduct the assessment. The quality of the included literature was evaluated on six indicators, including random allocation scheme, allocation scheme hiding, blind method, outcome data integrity, selective reporting of research results, and other biases. At the same time, PEDro scale (16) was used to evaluate the quality of the included studies, which included 11 items: the first item did not score, and each other item scored 1, a total of 10 points. A score of 3 and below is considered low quality, 4–5 is medium quality, 6–8 is high quality, and 9–10 is very high quality.

### 2.5 Statistical analysis

In this study, Review Manager5.3 software was used for analysis. Since the outcome indicators of the included literatures were continuous variables with the same measurement unit, mean difference (MD) and 95% confidence interval (CI) were used as the effect scale for analysis. The purpose of the meta-analysis was to derive the average effect size from different studies (17). In statistics, *p*-values are based on tests of significance, it is the probability that the event will happen by chance if the null hypothesis is true. The *p*-value is a number between 0 and 1 that the researcher interprets when deciding whether to reject or retain the null hypothesis (18). When *p* < 0.01, the result is highly significant. When 0.01 ≤ *p* < 0.05, there was a significant difference between the experimental group and the control group, and the results of meta-analysis were statistically significant. Q test is often used to evaluate heterogeneity. When *p* > 0.1, it indicates no heterogeneity (19). The variance can be used to describe the extent of variability in effect across studies (19, 20), it ranges from 0 to 100%. The Cochrane Handbook provides a rough yet widely used rule to interpret this measure: *I*^2^ ≤ 40% may indicate unimportant heterogeneity, 30% ≤ *I*^2^ ≤ 60% may represent moderate heterogeneity, 50% ≤ *I*^2^ ≤ 90% may represent substantial heterogeneity, and 75% ≤ *I*^2^ ≤ 100% implies considerable heterogeneity (21). When there is heterogeneity among the included studies, in which case, the random effects model was used for meta-analysis, and sensitivity analysis and subgroup analysis were performed to determine the source of heterogeneity. Funnel plot was used to analyze publication bias. This study mainly discusses the rehabilitation effect of different mind–body exercises on stroke patients.

## 3 Results

### 3.1 Literature search results

**Figure 1:**
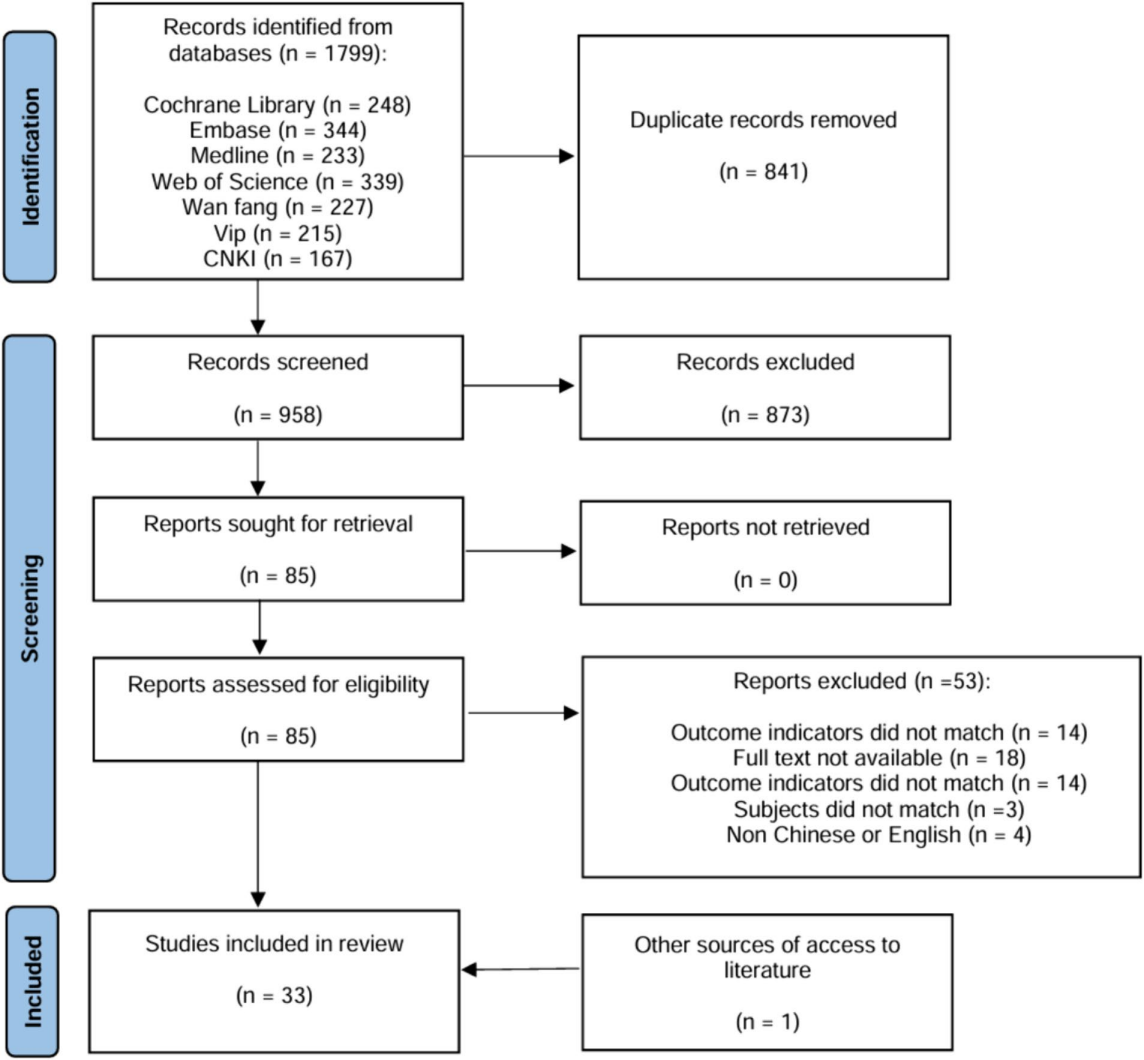
Screening diagram of included references.

Through the search of various databases, a total of 1799 articles were obtained. After importing the literature management software Endnote 20 to remove duplicate literatures, a total of 958 literatures were included. After reading the titles and abstracts of the literatures, 873 irrelevant literatures were excluded and 85 were left after preliminary screening. After further reading the full text, a total of 32 (22–54) RCT literatures were included in the meta-analysis. Furthermore, an additional literature was acquired through alternative methods, and a total of 33 literatures were ultimately included (Figure 1).

### 3.2 Basic features of the included literature

The basic characteristics of the included literatures are shown in Tables 1, 2. A total of 33 literatures (22–54) were included, including 1985 stroke patients, including 1,019 patients in the experimental group and 966 patients in the control group. Published from 2009 to 2024. A total of three mind–body exercises are involved, including 16 items of Tai Chi (22, 23, 27, 28, 30–34, 36, 38, 42, 44, 46, 49, 52), 15 items of Qigong (Baduanjin) (24, 25, 35, 37, 39–41, 43, 44, 47, 48, 50, 51, 53, 54) and 2 items of Yoga (26, 29).

**Table 1:** Summary of the included studies.

Author and year	Country	Total simple	M/F	Age (EG/CG)	Course of disease (EG/CG)
Au-Yeung 2009 (22)	Hong Kong	114	66/48	61.7 ± 10.5/65.9 ± 10.7	>6mon
Liu 2009 (23)	China	48	25/23	52.13 ± 14.13/53.51 ± 12.63	17.65 ± 5.34d/18.73 ± 8.78d
Bai 2011 (24)	China	60	42/18	53.7 ± 4.5/51.3 ± 7.5	43.2 ± 6.53d/38.5 ± 6.12d
Cai 2011 (25)	China	60	43/17	60.27 ± 10.48/61.27 ± 7.42	≥6mon
Schmid 2012 (26)	USA	47	30/28	63.9 ± 8.7/60.2 ± 8.9	>6mon
Yang 2013 (27)	China	100	66/34	54.3 ± 13.8/55.2 ± 14.6	26–63d
Xv 2014 (28)	China	80	38/42	60.14 ± 10.25/48.23 ± 12.32	47.34 ± 22.56d/45.21 ± 25.42d
Immink 2014 (29)	South Australia	22	9/13	56.1 ± 13.6/63.2 ± 17.4	81.6 ± 77.5 m/23.3 ± 12.5 m
Zheng 2015 (30)	China	106	58/48	59 ± 13/60 ± 12	First or multiple
Zhou 2015 (31)	China	22	Unknown	35–70	<6mon
Kim 2015 (32)	Korea	22	13/9	53.45 ± 11.54/55.18 ± 10.20	Unknown
Fu 2016 (33)	China	60	37/23	59.7 ± 7.6/60.3 ± 8.4	≤3mon
Yang 2016 (34)	China	60	41/19	60.71 ± 7.32/58.56 ± 8.52	First
Tian 2017 (35)	China	60	36/24	54.3 ± 4.7/53 ± 4.3	Unknown
Zhao 2017 (36)	China	60	39/21	53.85 ± 11.69/51.38 ± 14.83	First, steady within 1w
Cui 2018 (37)	China	43	27/16	53.67 ± 12.98/55.33 ± 14.32	47.32 ± 16.83d/45.84 ± 14.12d
Li 2018 (38)	China	60	33/27	71.03 ± 8.21/71.06 ± 8.33	110.7 ± 13.69d/102.60 ± 13.8d
Ding 2019 (39)	China	113	64/49	55.37 ± 4.71/56.32 ± 3.17	6.72 ± 2.36w/7.11 ± 1.59w
Wei 2019 (40)	China	80	38/42	56.1 ± 9.2/58.7 ± 10.3	15.4 ± 3.3mon/13.0 ± 2.3mon
Xie 2019 (41)	China	40	25/15	51.10 ± 12.92/53.95 ± 13.00	3.2 ± 1.44mon/3.60 ± 1.57mon
Zhang 2020 (42)	China	60	33/27	68.5 ± 9.4/65.5 ± 10.1	80.3 ± 30.4d/76.6 ± 31.9d
Liu 2021 (43)	China	60	21/39	57.58 ± 5.71/56.85 ± 7.47	15.42 ± 5.65w/15.61 ± 6.11w
Song 2021 (44)	Korea	34	21/13	58.72 ± 17.13/57.18 ± 10.65	7.58 ± 5.98/10.94 ± 8.50
Zhou 2021 (45)	China	70	42/28	69.1 ± 8.5/69.5 ± 8.3	1.91 ± 0.73mon/1.88 ± 0.84mon
He 2022 (46)	China	55	43/12	62.96 ± 8.98/62.50 ± 10.73	Unknown
Ji 2022 (47)	China	90	55/35	69.32 ± 5.94/68.42 ± 6.85	49.36 ± 7.48d/48.92 ± 6.37d
Liu 2022 (48)	China	43	27/16	59.19 ± 4.69/57.73 ± 5.55	8.08 ± 2.99w/9.07 ± 3.61w
Tang 2022 (49)	China	67	41/26	54.9 ± 13.1/56.5 ± 11.2	36.9 ± 12.2d/38.4 ± 10.8d
Chen 2023 (50)	China	80	50/30	62.33 ± 6.58/62.41 ± 6.63	12.28 ± 2.56mon/12.30 ± 2.66mon
Tang 2023 (51)	China	60	35/25	57.18 ± 7.71/57.14 ± 7.68	4.16 ± 1.65mon/4.13 ± 1.32mon
Wang 2023 (52)	China	17	13/4	49.11 ± 11.85/52.88 ± 11.79	67.22 ± 33.55d/52.50 ± 36.50d
Wang 2023 (53)	China	60	40/20	62.96 ± 6.18/62.94 ± 6.17	8.20 ± 3.66w/8.17 ± 3.65w
Chen 2024 (54)	China	42	34/8	52.86 ± 14.84/54.14 ± 12.30	210.57 ± 398.09d/183.67 ± 234.44d

M, Male; F, Female; EG, Experimental group; CG, Control group; d, day; w, week; mon, month.

**Table 2:** Experimental protocols and outcomes of the included studies.

Author and year	N/n	M/F	Intervention measure				Instructor	Location	Outcome index	Mark
			Mode	Cycle	Time	Frequency				
Au-Yeung 2009 (22)	E:59	33/26	Tai Chi	12w	1 h (3 h self-exercise)	1/w	Therapist	Community or Day care centers.	BBS TUGT	7
	C:55	33/22	Breathing and stretching exercises							
Liu 2009 (23)	E:24	14/10	Tai Chi	12w	30 min	7/w	Therapist	Home	BBS	6
	C:24	11/13	Routine rehabilitation training							
Bai 2011 (24)	E:30	20/10	Qigong (Baduanjin)	6w	20 min	14/w	Therapist	Hospital	BBS	7
	C:30	22/8	Balance function training							
Cai 2011 (25)	E:30	20/10	Qigong (Baduanjin)	12w	30 min	4-5/w	Nurse	Community	BI	7
	C:30	23/7	General health guidance							
Schmid 2012 (26)	E:37	20/22	Yoga	8w	1 h	16/w	Therapist	Home	BBS	7
	C:10	10/6	Routine care							
Yang 2013 (27)	E:50	35/15	Tai Chi	4w	45 min	6/w	Therapist	Hospital	BBS BI	7
	C:50	31/19	Balance function training							
Xv 2014 (28)	E:40	22/18	Tai Chi	12w	20 min	14/w	Therapist	Hospital	BBS	7
	C:40	16/24	Balance function training							
Immink 2014 (29)	E:11	6/5	Yoga	10w	90 min × 1 + 40 min × 6	7/w	Yoga teacher	Home	BBS	7
	C:11	3/8	Routine rehabilitation training							
Zheng2015 (30)	E:51	27/24	Tai Chi	48w	30 min	14/w	Therapist	Hospital	HAMD	7
	C:55	31/24	Routine rehabilitation training							
Zhou 2015 (31)	E:11		Tai Chi	4w	1 h	5/w	Therapist	Hospital	BBS FMA	7
	C:11		Routine rehabilitation training							
Kim 2015 (32)	E:11	7/4	Tai Chi	6w	1 h	2/w	Researcher	Hospital	TUGT	7
	C:11	6/5	General physical therapy							
Fu 2016 (33)	E:30	19/11	Tai Chi	8w	40 min	6/w	Therapist	Hospital	BBS	6
	C:30	18/12	Routine rehabilitation training							
Yang 2016 (34)	E:30	20/10	Tai Chi	8w	15 min	1/w	Therapist	Hospital	BI	7
	C:30	21/9	Walking ability training							
Tian 2017 (35)	E:30	17/13	Qigong (Baduanjin)	14w	30–40 min	2/w	Therapist	Hospital	BBS FMA	7
	C:30	19/11	Conventional treatment							
Zhao 2017 (36)	E:30	20/10	Tai Chi	8w	30 min	5/w	Therapist	Hospital	FMA HAMD BI	7
	C:30	19/11	Conventional treatment							
Cui 2018 (37)	E:24	15/9	Qigong (Baduanjin)	8w	45 min	3/w	Therapist	Hospital	FMA	7
	C:19	12/7	Conventional treatment							
Li 2018 (38)	E:30	17/13	Tai Chi	12w	1 h	7/w	Therapist	Hospital	FMA HAMD	7
	C:30	16/14	Drug therapy							
Ding 2019 (39)	E:57	33/24	Qigong (Baduanjin)	4w	20 min	10/w	Therapist	Hospital	BBS	7
	C:56	31/25	Balance function training							
Wei 2019 (40)	E:40	17/23	Qigong (Baduanjin)	6w	30 min	5/w	Therapist	Hospital	MBI	7
	C:40	21/19	Conventional treatment							
Xie 2019 (41)	E:20	13/7	Qigong (Baduanjin)	3w	50 min	3/w	Therapist	Hospital	BBS FMA BI	8
	C:20	12/8	General physical therapy							
Zhang 2020 (42)	E:30	17/13	Tai Chi	4w	20 min	14/w	Therapist	Hospital	BBS FMA	7
	C:30	16/14	Routine rehabilitation training							
Liu 2021 (43)	E:30	11/19	Qigong (Baduanjin)	4w	45 min	3/w	Therapist	Hospital	BBS FMA HAMD	7
	C:30	10/20	Drug therapy							
Song 2021 (44)	E:18	10/8	Tai Chi	24w	50 min	2/w	Tai chi instructor	Hospital	BBS	7
	C:16	11/5	Symptom management plan							
Zhou 2021 (45)	E:35	20/15	Qigong (Baduanjin)	12w	30 min	14/w	Therapist	Hospital	BBS FMA	7
	C:35	22/13	Routine rehabilitation training							
He 2022 (46)	E:29	23/6	Tai Chi	4w	40 min	4/w	Therapist	Hospital	BBS FMA	7
	C:26	20/6	Conventional treatment							
Ji 2022 (47)	E:45	26/19	Qigong (Baduanjin)	8w	15 min	6/w	Therapist	Hospital	FMA	7
	C:45	29/16	Routine rehabilitation training							
Liu 2022 (48)	E:21	14/7	Qigong (Baduanjin)	4w	30 min	12/w	Therapist	Hospital	BBS TUGT	8
	C:22	13/9	Conventional treatment							
Tang 2022 (49)	E:33	21/12	Tai Chi	8w	15 min	6/w	Therapist	Hospital	BBS FMA TUGT MBI	7
	C:34	20/14	Conventional treatment							
Chen 2023 (50)	E:40	24/16	Qigong (Baduanjin)	24w	40 min	6-10/w	Therapist	Hospital	FMA MBI	7
	C:40	26/14	Routine rehabilitation training							
Tang 2023 (51)	E:30	17/13	Qigong (Baduanjin)	8w	15 min	6/w	Therapist	Hospital	FMA HAMD	7
	C:30	18/12	Drug therapy							
Wang 2023 (52)	E:9	7/2	Tai Chi	4w	30 min	7/w	Therapist	Hospital	FMA MBI	7
	C:8	6/2	Conventional treatment							
Wang 2023 (53)	E:30	20/10	Qigong (Baduanjin)	4w	20 min	6/w	Therapist	Hospital	BBS FMA	7
	C:30	20/10	Conventional treatment							
Chen 2024 (54)	E:21	17/4	Qigong (Baduanjin)	4w	45 min	6/w	Therapist	Hospital	BBS FMA MBI	7
	C:21	17/4	Routine rehabilitation training							

BBS, Berg Balance Scale; FMA, Fugl-Meyer; TUGT, Timed Up-and-go Test; HAMD, Hamilton Depression Scale; BI, Barthel Index Rating Scale; MBI, Modified Barthel Index Rating Scale.

There was no statistical difference in the comparison of general data between the experimental group and the control group in each RCTS, which was comparable. There was no statistical difference in the comparison of general data between the experimental group and the control group in each RCTS, which was comparable.

### 3.3 Quality assessment of included literature

**Figure 2:**
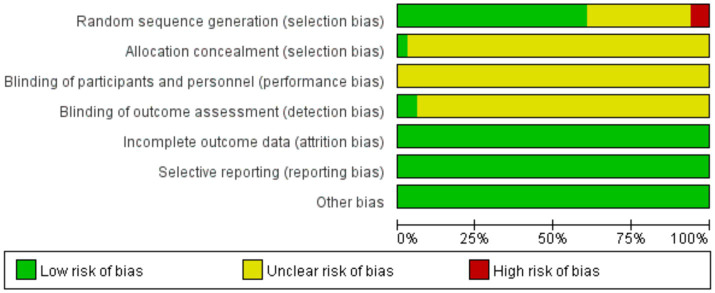
Risk shift diagram.

In the analysis of 33 studies (22–54), all studies reported the baseline condition of patients. Among them, 4 studies (22, 29, 46, 48) utilized computer randomization and were evaluated as low risk. Additionally, 16 studies (24, 25, 31, 36, 39–45, 47, 49, 51–54) employed the random number table method and were also evaluated as low risk. One study (23) randomized participants based on discharge order, while another study (33) used randomization according to sex, age, and lesion type. The remaining studies mentioned randomization without specifying the method. Only a study (44) mentioned single blindness. Only one study (48) reported allocation concealment and was evaluated as low risk in this aspect. Similarly, only one study (41) blinded outcome evaluators and was considered low risk in this regard. All studies had complete outcome data, and explanations for any lost follow-up or withdrawals were provided, without impacting the outcomes, leading to them being evaluated as low risk. None of the studies exhibited selective reporting of results (Table 3 and Figure 2).

**Table 3:** Literature quality evaluation.

Author and year	A	B	C	D	E	F	G
Au-Yeung 2009 (22)	Computer randomization	Unknown	Unknown	Unknown	Low	Low	Low
Liu 2009 (23)	Random according to discharge order	Unknown	Unknown	Unknown	Low	Low	Low
Bai 2011 (24)	Random number table method	Unknown	Unknown	Unknown	Low	Low	Low
Cai 2011 (25)	Random number table method	Unknown	Unknown	Unknown	Low	Low	Low
Schmid 2012 (26)	Random	Unknown	Unknown	Unknown	Low	Low	Low
Yang 2013 (27)	Random	Unknown	Unknown	Unknown	Low	Low	Low
Xv 2014 (28)	Random	Unknown	Unknown	Unknown	Low	Low	Low
Immink 2014 (29)	Computer randomization	Unknown	Unknown	Unknown	Low	Low	Low
Zheng 2015 (30)	Random	Unknown	Unknown	Unknown	Low	Low	Low
Zhou 2015 (31)	Random number table method	Unknown	Unknown	Unknown	Low	Low	Low
Kim 2015 (32)	Random	Unknown	Unknown	Unknown	Low	Low	Low
Fu 2016 (33)	Random according to sex, age and lesion type	Unknown	Unknown	Unknown	Low	Low	Low
Yang 2016 (34)	Random	Unknown	Unknown	Unknown	Low	Low	Low
Tian 2017 (35)	Random	Unknown	Unknown	Unknown	Low	Low	Low
Zhao 2017 (36)	Random number table method	Unknown	Unknown	Unknown	Low	Low	Low
Cui 2018 (37)	Random	Unknown	Unknown	Unknown	Low	Low	Low
Li 2018 (38)	Random	Unknown	Unknown	Unknown	Low	Low	Low
Ding 2019 (39)	Random number table method	Unknown	Unknown	Unknown	Low	Low	Low
Wei 2019 (40)	Random number table method	Unknown	Unknown	Unknown	Low	Low	Low
Xie 2019 (41)	Random number table method	Unknown	Unknown	Blind	Low	Low	Low
Zhang 2020 (42)	Random number table method	Unknown	Unknown	Unknown	Low	Low	Low
Liu 2021 (43)	Random	Unknown	Unknown	Unknown	Low	Low	Low
Song 2021 (44)	Random number table method	Unknown	Single blind	Unknown	Low	Low	Low
Zhou 2021 (45)	Random number table method	Unknown	Unknown	Unknown	Low	Low	Low
He 2022 (46)	Computer randomization	Unknown	Unknown	Unknown	Low	Low	Low
Ji 2022 (47)	Random number table method	Unknown	Unknown	Unknown	Low	Low	Low
Liu 2022 (48)	Computer randomization	An opaque envelope	Unknown	Unknown	Low	Low	Low
Tang 2022 (49)	Random number table method	Unknown	Unknown	Unknown	Low	Low	Low
Chen 2023 (50)	Random	Unknown	Unknown	Unknown	Low	Low	Low
Tang 2023 (51)	Random number table method	Unknown	Unknown	Unknown	Low	Low	Low
Wang 2023 (52)	Random number table method	Unknown	Unknown	Unknown	Low	Low	Low
Wang 2023 (53)	Random number table method	Unknown	Unknown	Unknown	Low	Low	Low
Chen 2024 (54)	Random number table method	Unknown	Unknown	Unknown	Low	Low	Low

A, Random sequence generation; B, Allocation hiding; C, Both subjects and interveners were blinded; D, Blind the outcome evaluators; E, Outcome indicator data integrity; F, Selective reporting of results; G, Other migration sources.

The PEDro scores of the included studies ranged from 6 to 8 points, indicating that these studies were of high quality.

### 3.4 Results of meta-analysis

#### 3.4.1 Influence of mind–body exercise on balance ability of stroke patients (BBS index)

**Figure 3:**
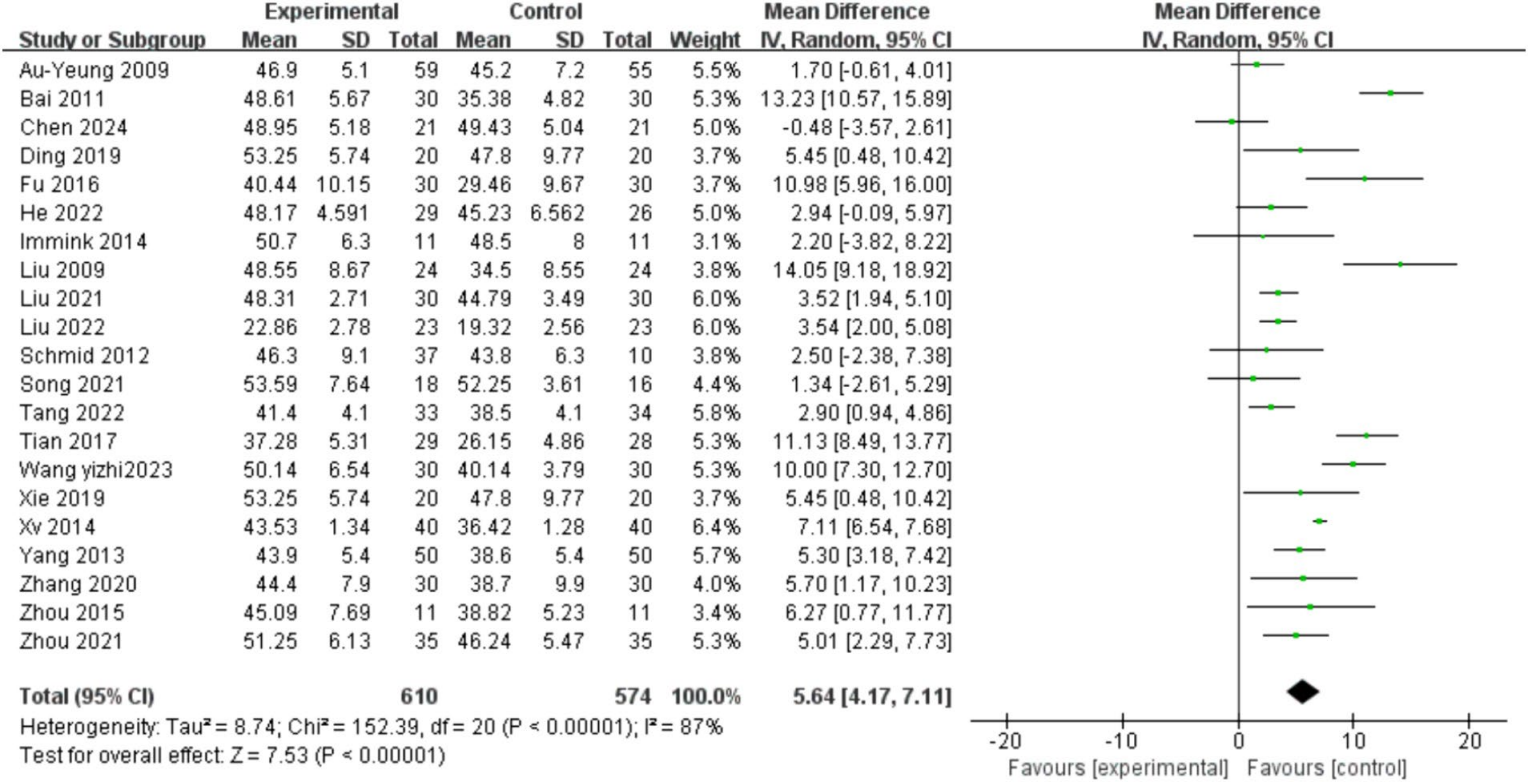
Effects of mind–body exercise on balance ability of stroke patients.

Among the included literatures, a total of 21 literatures (22–24, 26–29, 31, 33, 35, 39, 41–45, 47, 48, 52, 53) reported the effects of mind–body exercise on the balance ability of stroke patients, involving a total of 1,267 stroke patients. The results of meta-analysis (Figure 3) showed that the total effect size and 95%CI of the combined study were 5.64 [4.17, 7.11], *p* < 0.00001. The results showed that the balance ability increased significantly after exercise intervention, which was significantly different from the control group. Heterogeneity test (*I*^2^ = 87%), *I*^2^ > 75% indicated significant heterogeneity. A random effects model was selected for meta-analysis, and the source of heterogeneity was explored.

**Figure 4:**
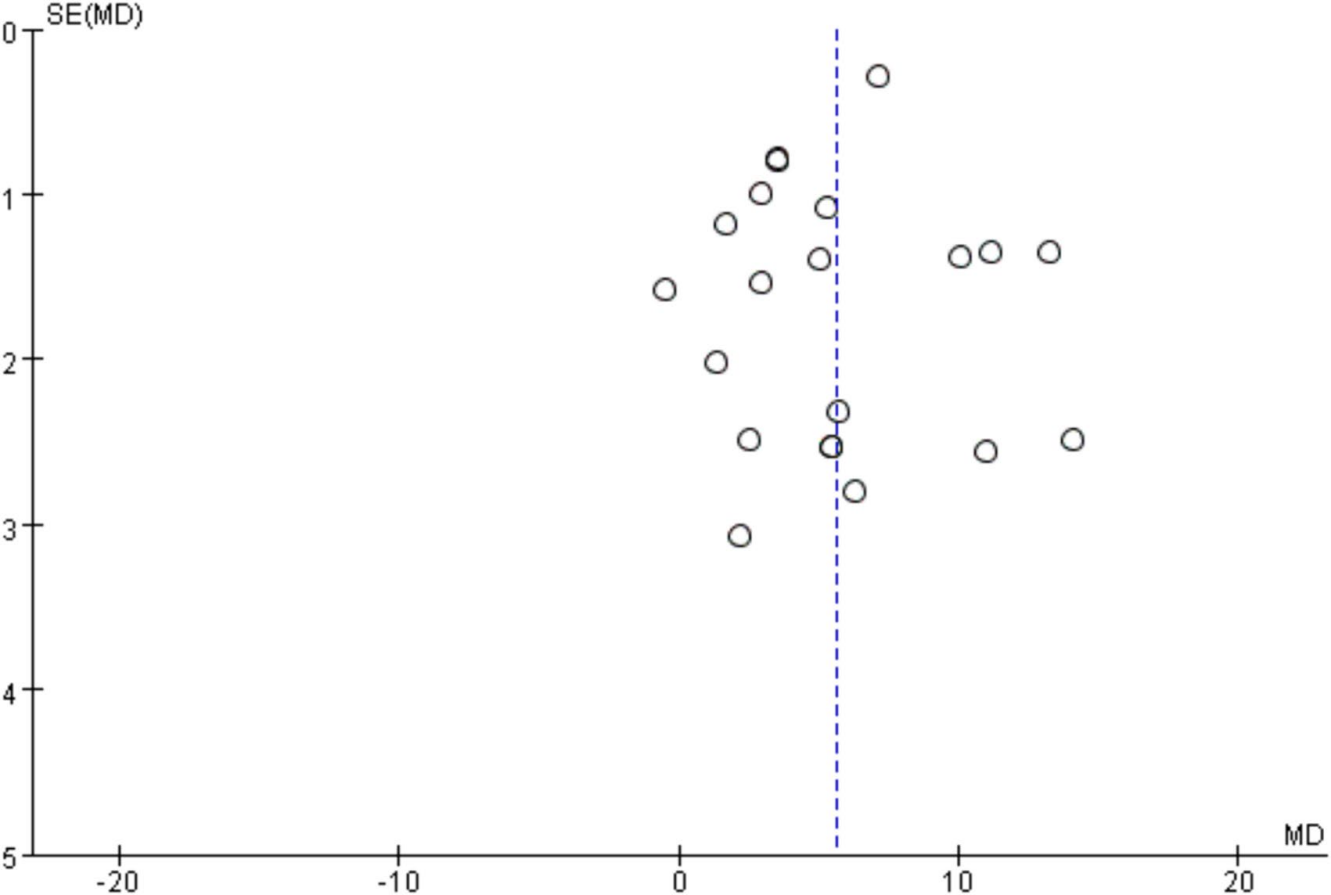
Funnel diagram of the influence of mind–body exercise on balance ability of stroke patients.

In order to explore the sources of heterogeneity, sensitivity analysis was used in the overall study to exclude the included studies one by one and assess the impact of each study on the index of balance ability. The results of meta-analysis showed that there was little difference in heterogeneity among all studies, the exclusion of one article had little impact on the index of balance ability, and the results of meta-analysis were stable. The traditional funnel plot was used to test publication bias, and the funnel plot could form a good left–right symmetric distribution without significant publication bias (Figure 4).

In order to further explore the sources of heterogeneity, subgroup analysis was conducted from four aspects: intervention mode, intervention cycle, intervention frequency, and intervention time (Table 4). In terms of intervention methods, Qigong (Baduanjin) has a significant effect on improving the balance function of stroke patients; In terms of intervention cycle, intervention for >4 weeks had a significant effect on improving the balance function of stroke patients; In terms of intervention time, 15–40 min of intervention has a significant effect on improving the balance function of stroke patients. In terms of intervention frequency, 6–10 interventions per week have a significant effect on improving balance function in stroke patients.

**Table 4:** Subgroup analysis of effects of mind–body exercise on balance ability in stroke patients.

Influencing factor	Number of studies	MD [95%CI]	Heterogeneity	
			*I*^2^ (%)	*p*-value
**Mode**				
Tai Chi	10	5.42 [3.40, 7.43]	85	<0.00001
Qigong (Baduanjin)	9	6.34 [3.53, 9.14]	91	<0.00001
Yoga	2	2.23 [−1.41, 6.17]	0	0.22
**Cycle (w)**				
≤4	10	4.59 [2.92, 6.26]	71	<0.00001
>4	11	6.56 [4.27, 8.85]	89	<0.00001
**Time (min)**				
15–40	12	7.44 [5.56, 9.32]	88	<0.00001
>40	9	2.99 [1.58, 4.41]	43	<0.0001
**Frequency**				
<6	9	3.76 [1.58, 5.94]	82	0.0007
6–10	6	8.06 [4.93, 11.20]	75	<0.00001
>10	6	6.37 [3.84, 8.90]	89	<0.00001

#### 3.4.2 Effects of mind–body exercise on motor ability of stroke patients (FMA index)

Among the included literatures, a total of 16 literatures (21 research reports) (31, 35–38, 41–46, 48–53) reported the impact of mind–body exercise on the motor ability of stroke patients, involving a total of 1,120 stroke patients.

##### 3.4.2.1 Upper limb motor function (FMA-UE index)

**Figure 5:**
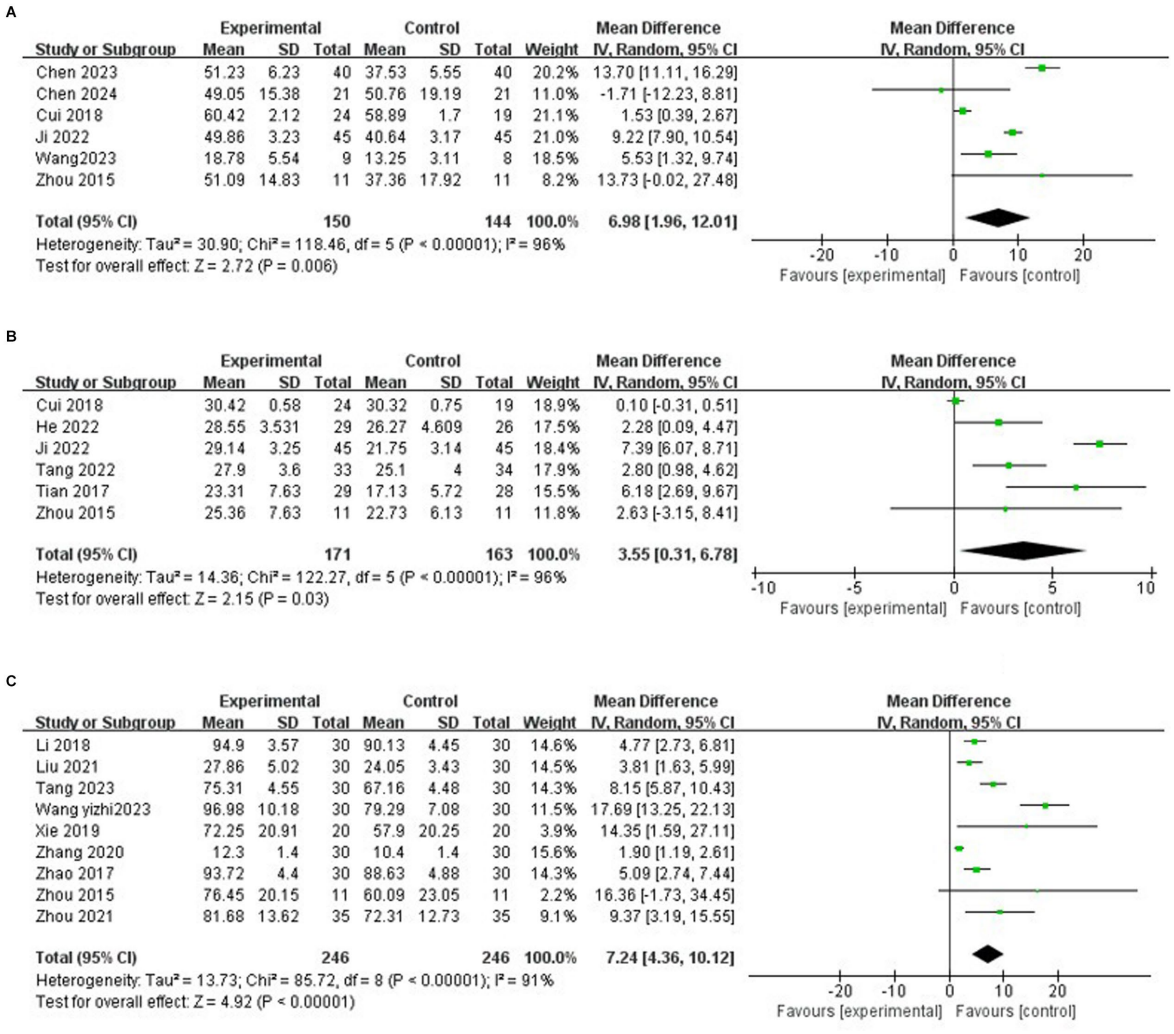
**(A)** Effects of mind–body exercise on upper limb motor ability in stroke patients. **(B)** Effects of mind–body exercise on lower limb motor ability of stroke patients. **(C)** Effects of mind–body exercise on the motor ability of stroke patients.

Six studies reported the effects of mind–body exercise on upper limb motor ability in stroke patients. The results of meta-analysis (Figure 5A) showed that the total effect size and 95%CI of the combined study were 6.98 [1.96, 12.01], *p* = 0.006 < 0.01. The results showed that the exercise ability increased significantly after the intervention, and there was a significant difference compared with the control group. Heterogeneity test (*I*^2^ = 96%), *I*^2^ > 75% indicated significant heterogeneity. A random effects model was selected for meta-analysis, and the source of heterogeneity was explored.

To explore the sources of heterogeneity, sensitivity analysis was used in the overall study to exclude the included studies one by one and assess the impact of each study on the indicators of motor ability. The study showed that after the article by Cui et al. (37) was excluded, the heterogeneity was significantly reduced (*I*^2^ = 78%), that mean, Cui et al. (37) was the source of heterogeneity of the influence of mind–body exercise on the upper limb motor ability of stroke patients.

##### 3.4.2.2 Lower limb motor function (FMA-LE index)

Among them, 6 studies reported the effects of mind–body exercise on lower limb motor ability of stroke patients. The results of meta-analysis (Figure 5B) showed that the total effect size and 95%CI of the combined study were 3.55 [0.31, 6.78], *p* = 0.03 < 0.05. The results showed that the exercise ability increased after the mind–body exercise intervention, and there was a significant difference compared with the control group. Heterogeneity test (*I*^2^ = 96%), *I*^2^ > 75% indicated significant heterogeneity. A random effects model was selected for meta-analysis, and the source of heterogeneity was explored.

To explore the sources of heterogeneity, sensitivity analysis was used in the overall study to exclude the included studies one by one and assess the impact of each study on the indicators of motor ability. The study showed that after the article by Cui et al. (32) was excluded, the heterogeneity was reduced (*I*^2^ = 84%), that is, Cui et al. (32) was the source of heterogeneity of the influence of mind–body exercise on lower limb motor ability of stroke patients.

##### 3.4.2.3 Motion function (FMA index)

Among them, 9 studies reported the effect of mind–body exercise on the overall motor ability of stroke patients. The results of meta-analysis (Figure 5C) showed that the total effect size and 95%CI of the combined study were 7.24 [4.36, 10.12], *p* < 0.00001. The results showed that the exercise ability increased significantly after the intervention, and there was a significant difference compared with the control group. Heterogeneity test (*I*^2^ = 91%), *I*^2^ > 75% indicated significant heterogeneity. A random effects model was selected for meta-analysis, and the source of heterogeneity was explored.

To explore the sources of heterogeneity, sensitivity analysis was used in the overall study to exclude the included studies one by one and assess the impact of each study on the indicators of motor ability. Studies have shown that there is little difference in heterogeneity among different studies, and the exclusion of a certain article has little effect on the overall motor ability of stroke patients.

#### 3.4.3 Influence of mind–body exercise on walking ability of stroke patients (TUGT index)

**Figure 6:**
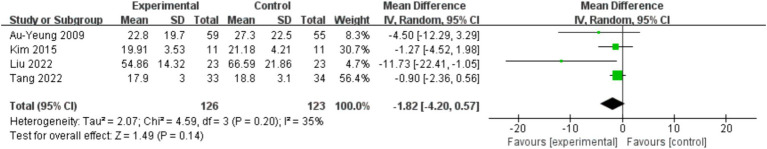
Effects of mind–body exercise on walking ability of stroke patients.

Among the included literatures, a total of 4 literatures (22, 32, 47, 48) reported the effects of physical and mental exercise on walking ability of stroke patients, involving a total of 248 stroke patients. The results of meta-analysis (Figure 6) showed that the total effect size and 95%CI of the combined study were − 1.82 [−4.20, 0.57], *p* = 0.14. The results showed that the walking ability was improved after exercise intervention, but there was no significant difference compared with the control group. Heterogeneity test (*I*^2^ = 35%), *I*^2^ < 40% indicates that the heterogeneity is small, the fixed effect model was selected for meta-analysis, and the source of heterogeneity was explored.

In order to explore the sources of heterogeneity, sensitivity analysis was used in the overall study to exclude the included studies one by one and assess the impact of each study on the index of balance ability. Studies have shown that after the article by Liu et al. (47) was excluded, the heterogeneity was significantly reduced (*I*^2^ = 0%), that mean, Liu et al. (47) was the main source of heterogeneity in the impact of mind–body exercise on the walking ability of stroke patients.

#### 3.4.4 Effects of mind–body exercise on depression in stroke patients (HAMD index)

**Figure 7:**
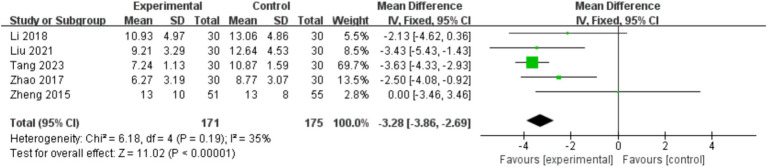
Effects of mind–body exercise on depression in stroke patients.

Among the included literatures, a total of 5 literatures (30, 36, 38, 43, 50) reported the effects of mind–body exercise on post-stroke depression, involving a total of 346 stroke patients. The results of meta-analysis (Figure 7) showed that the total effect size and 95%CI of the combined study were −3.28 [−3.86, −2.69], *p* < 0.00001. The results showed that depressive mood improved significantly after exercise intervention, and there was a significant difference compared with the control group. Heterogeneity test (*I*^2^ = 35%), *I*^2^ < 40% indicates that the heterogeneity is small, the fixed effect model was selected for meta-analysis, and the source of heterogeneity was explored.

In order to explore the sources of heterogeneity, sensitivity analysis was used in the overall study to exclude the included studies one by one and assess the impact of each study on the index of balance ability. The study showed that after removing the article of Zheng et al. (30), the heterogeneity was significantly reduced (*I*^2^ = 0%), that mean, Zheng et al. (30) was the main source of heterogeneity in the influence of mind–body exercise on depression in stroke patients.

#### 3.4.5 Effects of mind–body exercise on quality of life of stroke patients (MBI, BI index)

**Figure 8:**
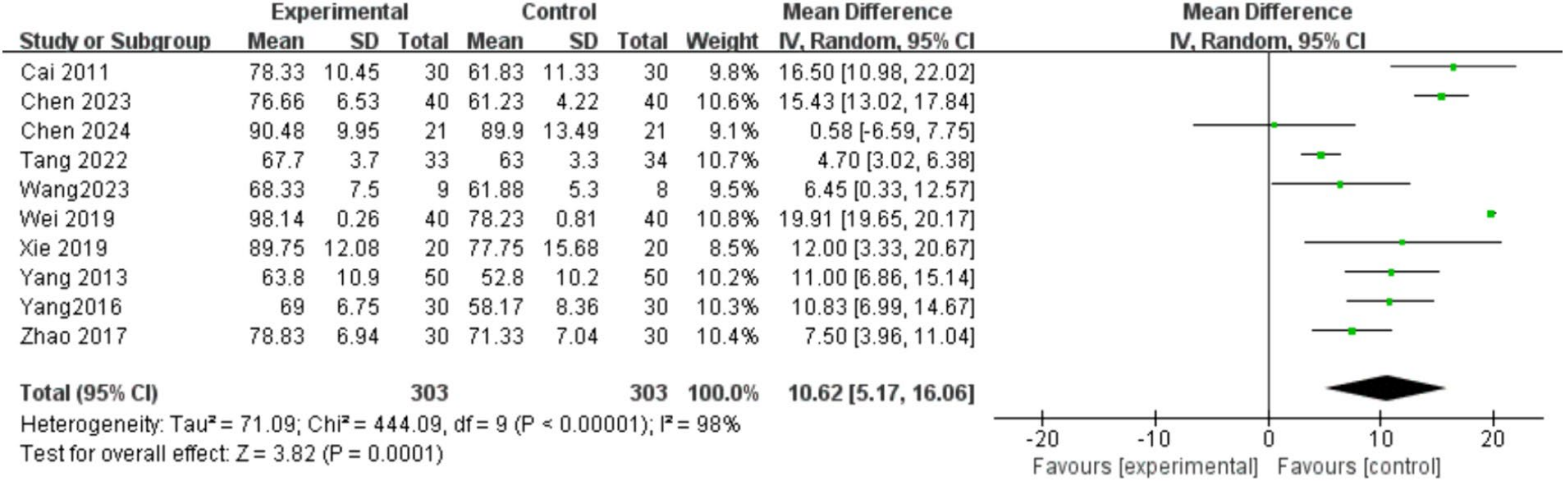
Effects of mind–body exercise on quality of life of stroke patients.

Among the included studies, a total of 10 articles (25, 27, 34, 36, 40, 41, 48, 49, 51, 53) reported the effects of mind–body exercise on the quality of life of stroke patients. The results of meta-analysis (Figure 8) showed that the total effect size and 95%CI of the combined study were 10.62 [5.17, 16.06], *p* = 0.0001 < 0.01. The results showed that the quality of life increased significantly after the mind–body exercise intervention, and there was a significant difference compared with the control group. Heterogeneity test (*I*^2^ = 98%), *I*^2^ > 75% indicated significant heterogeneity. A random effects model was selected for meta-analysis, and the source of heterogeneity was explored.

**Figure 9:**
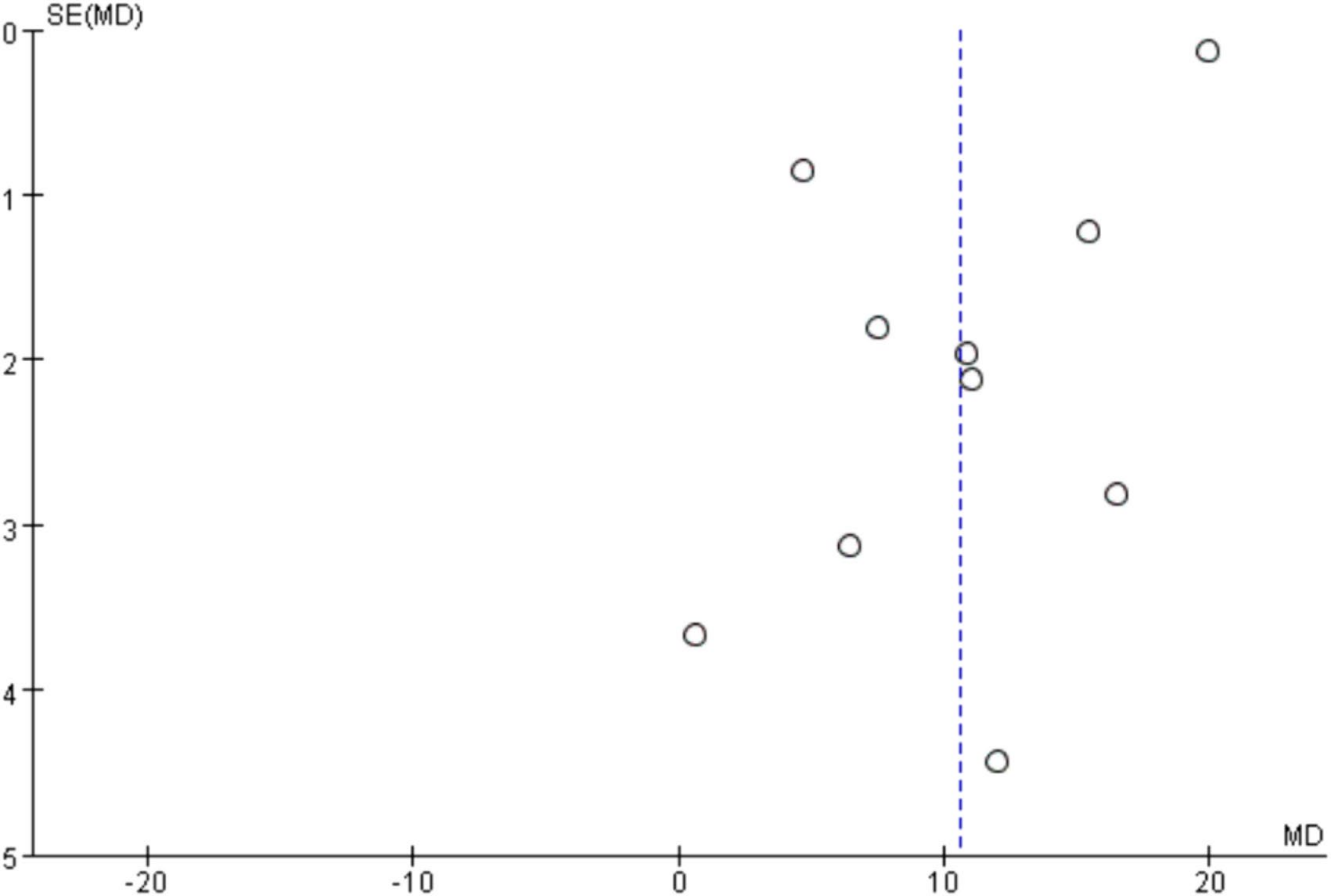
Funnel diagram of the influence of mind–body exercise on the quality of life of stroke patients.

To explore the sources of heterogeneity, sensitivity analysis was used in the overall study to exclude the included studies one by one and assess the impact of each study on the indicators of motor ability. Studies have shown that there is little difference in heterogeneity among all studies, and the exclusion of a certain article has little impact on the quality of life of stroke patients. The results of meta-analysis are stable, and the traditional funnel plot is used to test publication bias, and the funnel plot can form a good left–right symmetric distribution, and there is no obvious publication bias (Figure 9).

In order to further explore the sources of heterogeneity, subgroup analysis was conducted from four aspects: intervention mode, intervention cycle, intervention frequency, and intervention time (Table 5). In terms of intervention methods, Qigong (Baduanjin) has a significant effect on improving the quality of life of stroke patients; In terms of intervention cycle, intervention for >4 weeks has a significant effect on improving the quality of life of stroke patients; In terms of intervention time, 15–40 min of intervention had a significant effect on improving the quality of life of stroke patients. In terms of intervention frequency, 6–10 interventions per week have a significant effect on improving the quality of life of stroke patients.

**Table 5:** Subgroup analysis of effects of mind–body exercise on quality of life in stroke patients.

Influencing factor	Number of studies	MD [95%CI]	Heterogeneity	
			*I*^2^ (%)	*p*-value
**Mode**				
Tai Chi	5	7.92 [4.79, 10.87]	72	<0.00001
Qigong (Baduanjin)	5	13.88 [9.10, 18.67]	91	<0.00001
**Cycle (w)**				
≤4	4	7.67 [2.83, 12.51]	58	0.002
>4	6	12.46 [5.56, 19.37]	99	0.0004
**Time (min)**				
15–40	7	11.67 [5.22, 18.12]	98	0.0004
>40	3	7.98 [1.11, 14.84]	70	0.02
**Frequency**				
<6	7	10.37 [2.97, 17.77]	99	0.006
6–10	3	11.57 [6.63, 16.51]	78	<0.00001

## 4 Discussion

Meta-analysis has become a key tool for promoting rapid scientific progress. Through the integration and comprehensive analysis of scientific results from multiple studies, the effectiveness of scientific research can be greatly improved (55).

In this meta-analysis, BBS, FMA, and TUGT were employed to evaluate the physical function changes in stroke patients, encompassing balance function, motor function, and walking ability. HAMD was utilized to assess the mental health aspect, specifically focusing on alleviating depression. The changes of life quality were evaluated using MBI and BI. These metrics collectively facilitated an in-depth exploration of the impact of mind–body exercise interventions on the physical function, mental health, and quality of life of stroke patients.

### 4.1 Influence of mind–body exercise on the physical function of stroke patients

Post-stroke, individuals commonly face hemiplegia, upper and lower limb dysfunction, and compromised balance and motor control, leading to reduced walking ability and unstable gait, potentially triggering fear of falling or depressive symptoms. The meta-analysis revealed significant enhancements in balance and motor function with mind–body exercise, although improvements in walking function were less pronounced. Combining mind–body exercise with conventional rehabilitation therapy effectively boosted BBS scores and balance abilities in stroke patients, aligning with prior research (11, 56). Subgroup analysis highlighted Qigong (Baduanjin) as a promising rehabilitation therapy for enhancing BBS scores and balance function. Baduanjin, a traditional Chinese fitness regimen, is universally applicable and not only enhances musculoskeletal and neuromuscular functions but also fosters mental relaxation and breathing control, promoting patient satisfaction and exercise adherence (11). Modern medical studies (57) demonstrate that Baduanjin, rooted in “breath regulation,” enhances cortical function and the regulation mechanism of the cortical-ponto-cerebellar neural circuit, refining bodily movements and balance. Moreover, a duration of over 4 weeks of mind–body exercise was found to enhance patients’ balance, consistent with the findings of Li et al. (58) and Tan et al. (59). Additionally, 15–40 min of exercise sessions 6–10 times a week significantly improved balance function.

The FMA score, a primary scale assessing limb motor ability post-stroke, demonstrated higher scores in the experimental group than the control group, indicating improved upper and lower limb motor function and overall motor performance with mind–body exercise, consistent with previous studies (60, 61). However, there was a high degree of heterogeneity in the included literature on limb motor ability, so it was necessary to exclude specific studies to reduce heterogeneity. After excluding the study by Cui et al. (32), heterogeneity remained high, which may be attributed to differences in study populations, interventions, and numbers.

The TUGT test, a rapid assessment of functional walking ability, showed that stroke patients who underwent mind–body exercise rehabilitation showed improved walking ability, but did not show statistically significant differences compared with conventional rehabilitation. Due to the limited number of literatures on this outcome indicator, it is not possible to definitively determine whether mind–body exercise has an advantage in improving walking ability in stroke patients. It is hoped that more randomized controlled trials (RCTs) will be conducted in this area in the future. Mind–body exercises contain elements of physical and mental coordination, requiring concentration and breathing control. There is growing evidence that mind–body exercise has a positive impact on the effectiveness and safety of a variety of health conditions (60, 62, 63).

### 4.2 Influence of mind–body exercise on the mental health of stroke patients

Emotional disorders are one of the important indicators affecting the recovery of function in stroke patients (64). After stroke, they often face problems such as movement disorders and mobility difficulties, followed by a variety of life problems and expensive rehabilitation costs, which not only increase the burden of individuals and families, but also have a certain impact on their psychology, and then appear depression, anxiety and other conditions. HAMD depression scale and HAMD anxiety Scale are commonly used to evaluate the emotional state of patients in clinical practice. In this paper, depression index is selected to evaluate the rehabilitation effect of mind–body exercise on the mental health of stroke patients. The results of meta-analysis showed that, compared with conventional rehabilitation therapy, mind–body exercise had significant advantages in improving patients’ depression. However, due to its small sample size, it is still necessary to include more RCTS in the future to fully demonstrate the reliability of this result. At the same time, the relevant literature on anxiety indicators can be included to judge the improvement effect of physical and mental exercise on the anxiety of stroke patients.

### 4.3 Influence of mind–body exercise on the quality of life of stroke patients

The World Health Organization defines quality of life as an individual’s perception of their status in life by their standards, expectations and concerns in the larger context of the society in which they live. It can be said that quality of life is a subjective evaluation embedded in cultural, social and environmental contexts (65). In order to expand the sample size of the meta-analysis and enhance the quality and clinical reliability of the systematic study, this paper combined MBI and BI indicators to jointly evaluate the quality of life of stroke patients. The results showed that, compared with conventional rehabilitation therapy, mind–body exercise also had significant advantages in improving the quality of life of stroke patients, and the difference was statistically significant. Further subgroup analysis showed that Qigong (Baduanjin) was most likely to be the best rehabilitation therapy in improving the quality of life of stroke patients. At the same time, the quantitative analysis of this index found that mind–body exercise for more than 4 weeks, 6–10 times a week, 15–40 min each time significantly improved the quality of life of stroke patients.

### 4.4 Possible mechanism of action of mind–body exercise influencing stroke

For the improvement of the physical function of stroke patients, the reason may be that patients can promote the blood flow of the whole body through long-term stretching exercise and supplemented by breathing, which helps to establish the branch circulation of the brain and replace the damaged brain tissue, thus helping to re-cover the nerve pathway and improve the physical function of patients. In addition, the practice of Taijiquan requires attention and awareness, which requires the active participation of the brain to improve the tension of the central nervous system and strengthen the regulating role of the brain (66).

Improving the mental health of stroke patients. After Tai Chi intervention, several biomarkers associated with depression improved. Superoxide dismutase (SOD) is the most commonly mentioned antioxidant enzyme in depressive disorders, and Tai Chi has an antioxidant effect, which reduces the activity of SOD and thus reduces the level of depression (67). In addition, elevated inflammatory biomarkers are also common in people with depression. Interleukin is a commonly used protein and a well-known biomarker of inflammation, stress, and depression. Studies have shown that interleukin can be down-regulated after Tai Chi intervention (66, 68). In addition, an interesting explanation is that traditional mind–body exercises, such as Qigong, can be understood as an attempt to enhance proprioception, which can improve and regulate a person’s overall state and reduce anxiety by combining specific states of consciousness with posture, movement, and breathing control (67). The above is possible mechanisms for how mind–body exercises improve the psychological health of stroke patients.

### 4.5 Limitations and prospects

Its limitations are mainly manifested in the following aspects. First of all, the included literature only includes Chinese and English literature, which may cause some language deviation; Secondly, the geographical location of each study may affect the clinical significance and applicability. In addition, most of the included literatures did not use the blind method, which resulted in a decrease in the reliability of the studies to a certain extent. Finally, this study shows that it is feasible to perform tai chi exercises 6–10 times/week and 15–40 min each time during stroke recovery. However, large-scale applications are needed for special types of stroke patients.

In view of the above limitations, this study needs more high-quality clinical trials to standardize the optimal intervention time, form, frequency, intensity, standardized study design and intervention plan of mind–body exercise. Secondly, researchers should unify outcome evaluation indicators, and combine qualitative evaluation with quantitative evaluation to enrich outcome content. In addition, the shortcomings of methodology should be improved, the research process should be rigorous and standardized, and reliable evidence should be provided for the promotion and application of mind–body exercise intervention.

## 5 Conclusion

Existing evidence shows that mind–body exercise has significant advantages in the clinical rehabilitation of stroke patients, mainly in enhancing patients’ balance function, improving exercise ability, regulating depression, improving quality of life, etc. However, due to the lack of sufficient evidence, it is still unable to prove the role of mind–body exercise in improving the walking ability of stroke patients. Subgroup analysis results showed that Qigong (Baduanjin) training for more than 4 weeks, 6–10 times per week, 15–40 min each time can significantly improve balance function and quality of life in stroke patients. In this review, the two funnel plots are relatively symmetrical, which indicates that the possibility of publication deviation is small, and it has certain reliability and objectivity. Therefore, the exercise prescription obtained can be verified and applied to clinical practice to add a new exercise option for the rehabilitation of stroke patients. Based on the above discussion, mind–body exercise can be used as a supplement therapy to conventional rehabilitation therapy, which is not only low intensity, high safety, but also because it is not limited to the site, can be accepted by most people and accelerate the rehabilitation process of stroke.
