# Supplementary material for: Effects of mind–body exercise on physical ability, mental health and quality of life in stroke patients: a systematic review and meta-analysis
Source: Front Public Health. 2024 Dec 20;12:1432510. doi: 10.3389/fpubh.2024.1432510 (PMC11697286; doi:10.3389/fpubh.2024.1432510)
Supplement: Supplementary file 1 [file Table_1.DOCX]

Table 1.⎯ Search strategy in Web of Science

| Tab.1 Search strategy | |
| --- | --- |
| Order | Search query |
| 1 | (((((TS=(mind-body exercise)) OR TS=(Tai Chi)) OR TS=(Taiji)) OR TS=(Baduanjin)) OR TS=(Qigong)) OR TS=(Yoga) |
| 2 | (((((((((TS=(Stroke)) OR TS=(Cerebrovascular Accident)) OR TS=(CVA)) OR TS=(Cerebrovascular Apoplexy)) OR TS=(Brain Vascular Accident)) OR TS=(Cerebrovascular Stroke)) OR TS=(Apoplexy)) OR TS=(Cerebral Stroke)) OR TS=(Acute Stroke)) OR TS=(Acute Cerebrovascular Accident) |
| 3 | (((((((((TS=(balance capacity)) OR TS=(balance ability)) OR TS=(physical ability)) OR TS=(mental health)) OR TS=(motor function)) OR TS=(motor ability)) OR TS=(depression)) OR TS=(quality of life)) OR TS=(Health-Related Quality Of Life)) OR TS=(life quality) |
| 4 | ((((TS=(randomized)) OR TS=(randomized controlled trial)) OR TS=(clinical)) OR TS=(trial)) OR TS=(random) |
| 5 | #1 AND #2AND#3AND#4 |
